# Expression and mechanism of exosome-mediated A FOXM1 related long noncoding RNA in gastric cancer

**DOI:** 10.1186/s12951-021-00873-w

**Published:** 2021-05-10

**Authors:** Yan Zhang, Lin Chen, Xuanting Ye, Zhixiong Wu, Zeyu Zhang, Biaofeng Sun, Hong Fu, Chuangang Fu, Xiaofei Liang, Hong Jiang

**Affiliations:** 1 Department of Oncology, Tongji Hospital, Tongji University School of Medicine, No. 389, Putuoxincun Rd., Shanghai, 200065 China; 2 Department of Colorectal Surgery, Department of General Surgery, Shanghai East Hospital, Tongji University School of Medicine, 150 Jimo Road, Shanghai, 200120 China; 3 Shanghai University of Traditional Chinese Medicine, Shanghai, China; 4 Huzhou Lieyuan Medical Laboratory Company Ltd., No. 800, Rujiadian Rd., Huzhou, 313000 China

**Keywords:** Gastric cancer, Exosomes, FOXM1, Long non coding RNA, Biomarker

## Abstract

**Background:**

Forkhead box protein M1 (FOXM1) is an oncogene regulating tumor growth and metastasis. Exosome was suggested to mediate cell communication by delivering active molecules in cancers. However, the existence of FOXM1 in circulating exosomes and the role of exosome FOXM1 in gastric cancer (GC) were not clear. This study aims to investigate the potential role of FOXM1 related long noncoding RNA (FRLnc1) in exosomes in GC.

**Results:**

The prepared CD63 immunomagnetic beads (CD63-IMB) had the characteristics of good dispersity and high magnetic response. The isolated exosomes were presented with elliptical membranous particles under a transmission electron microscope (TEM), with the particle size of 89.78 ± 4.8 nm. Western blot (WB) results showed that the exosomes were rich in CD9 and CD81. The Dil-labeled exosomes were distributed around cytoplasm and nucleus of cells by imaging flow cytometry (IFC) analysis. The results of quantitative real-time PCR (qRT-PCR) revealed that the FRLnc1 expressions were up-regulated in GC cells, tumor tissues, and serum of GC patients. An obviously up-regulated FRLnc1 expression was found in serum exosomes of GC patients. Up-regulation of FRLnc1 expression was closely correlated to lymph node metastasis (LNM) and TNM stage with the combination of relevant clinicopathological parameter analysis. The in vitro functional analyses demonstrated that FRLnc1 knockdown by RNA interference suppressed cell proliferation and migration in HGC-27 cells, whereas FRLnc1 overexpression promoted cell proliferation and migration in MKN45 cells. After exosome treatment, the FRLnc1 expression was significantly increased in MKN45 cells, and the MKN45 cells showed increased ability of proliferation and migration.

**Conclusion:**

GC cells-derived exosomes played roles in promoting the growth and metastasis of GC by transporting FRLnc1, suggesting that FRLnc1 in the exosomes may be a potential biomarker for the diagnosis and treatment of GC. The delivery of FRLnc1 by the exosomes may provide a new way for the treatment of GC.

*Trial registration* 2020-KYSB-094. Registered 23 March 2020—Retrospectively registered

**Graphic abstract:**

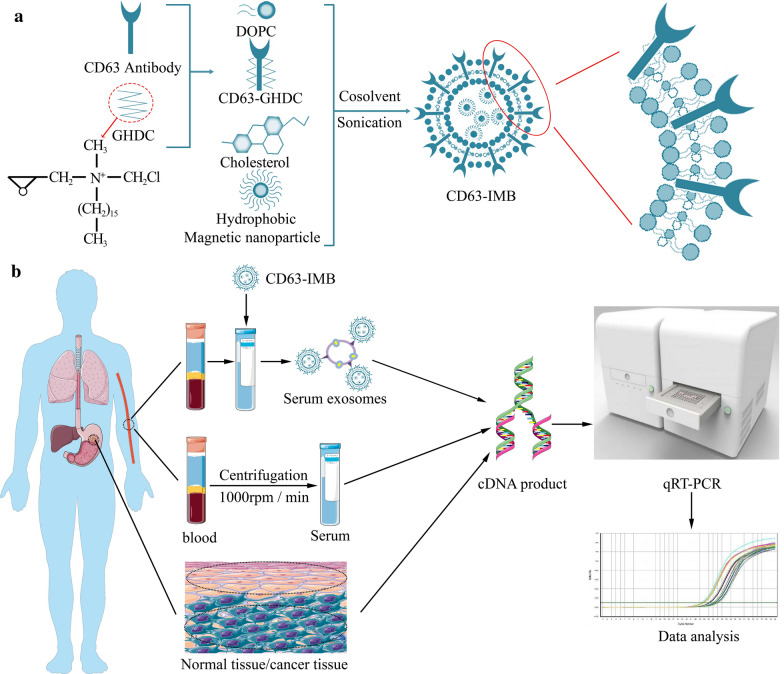

**Supplementary Information:**

The online version contains supplementary material available at 10.1186/s12951-021-00873-w.

## Background

Gastric cancer (GC) is a highly heterogeneous disease. It is the second leading cause of cancer death in the world. It is also a common gastrointestinal malignant tumor and ranks the fourth cause of death of malignant tumors [1]. Despite the recent advances in chemotherapy, radiotherapy and surgery for the treatment of GC, the survival rate of GC patients was still unsatisfactory [2, 3]. It was reported that the lack of appropriate molecular biomarkers was one of the main reasons for the low overall survival rate of GC patients, as a result, without effectively early diagnosis, most GC patients missed the best opportunity for surgical treatment [4]. Therefore, the discovery of a new specific biomarker was of great significance for the early diagnosis, prognosis evaluation and treatment of GC.

Forkhead box protein M1 (FOXM1), a transcription factor characterized by a 100 amino acid winged-helix DNA binding domain, plays im-portant roles in the regulation of oncogenesis and tumor progression in malignancies, forkhead box protein M1 (FOXM1) is one of the members of the family of evolutionarily conserved transcriptional factors characterized by the presence of a DNA-binding domain called the forkhead box or winged helix domain [5–7]. FOXM1, a transcriptional factor over expressing in majority of tumors, plays crucial roles in cancer cell proliferation, migration, and apoptosis [8, 9]. It has been reported that alterations in FOXM1 signaling pathways were associated with the occurrence of various tumors, and FOXM1 was suggested to be a prognostic predictor of GC [6, 10, 11]. Furthermore, it was found that FOXM1 over-expression could promote cell growth and angiogenesis [12]. Previous studies have confirmed that FOXM1 was an important oncogene for the occurrence of GC, and played a role in promoting the expression of FRLnc1, an FOXM1 related long noncoding RNA (lncRNA), indicating that FRLnc1 expression may be an independent prognostic marker for predicting survival in GC patients [6, 13].

The exosomes, nanoscale bilayer membrane vesicles secreted by living cells, were important mediators for cell signal transduction, and had various biological effects in promoting tumor angiogenesis and metastasis. The exosomes contained proteins, DNA and mRNA, as well as noncoding RNA, including microRNA, lncRNA and circular RNA [14, 15]. Studies have found that almost all cells can secrete exosomes, which can transport their bioactive substances, such as proteins and nucleic acids, to different cells, resulting in changes in the genetic information and cell functions of the recipient cells [16], various evidences indicate that exosomes are involved in the occurrence, growth, metastasis and drug resistance of tumors [17]. Studies have found that exosomes also contain lncRNA. Exosomes can protect lncRNA and make it stably exist in the blood [18]. At the same time, the expression level of exosomes lncRNA can help identify benign and malignant diseases [19]. Also exosome-mediated lncRNA plays an important role in tumor drug resistance [20]. Therefore, the study of exosomal lncRNA can provide important biomarkers for tumor diagnosis and treatment.

The present study aims to evaluate the role of FRLnc1 in exosomes as a biomarker for the diagnosis of GC through the detection of FRLnc1 expression in serum exosomes of GC patients. Specifically, quantitative real-time PCR (qRT-PCR) was used to detect the expression of FRLnc1 in serum exosomes, and the correlations of the up-regulation of FRLnc1 with lymph node metastasis (LNM) and TNM stage were analyzed with the combination of relevant clinicopathological parameters. The growth and migration abilities of gastric cancer cells with FRLnc1 overexpression or knockdown in vitro were evaluated by colony formation assay and transwell migration assay. Meanwhile, the exosomes containing FRLnc1 were isolated from SGC-7901 GC cells and purified, and the MKN45 GC cells with lowly-expressed FRLnc1 were treated with the exosomes containing FRLnc1 to further study the role of FRLnc1 in the exosomes in the occurrence and development of GC.

## Results

### Material characterization test

**Fig. 1:**
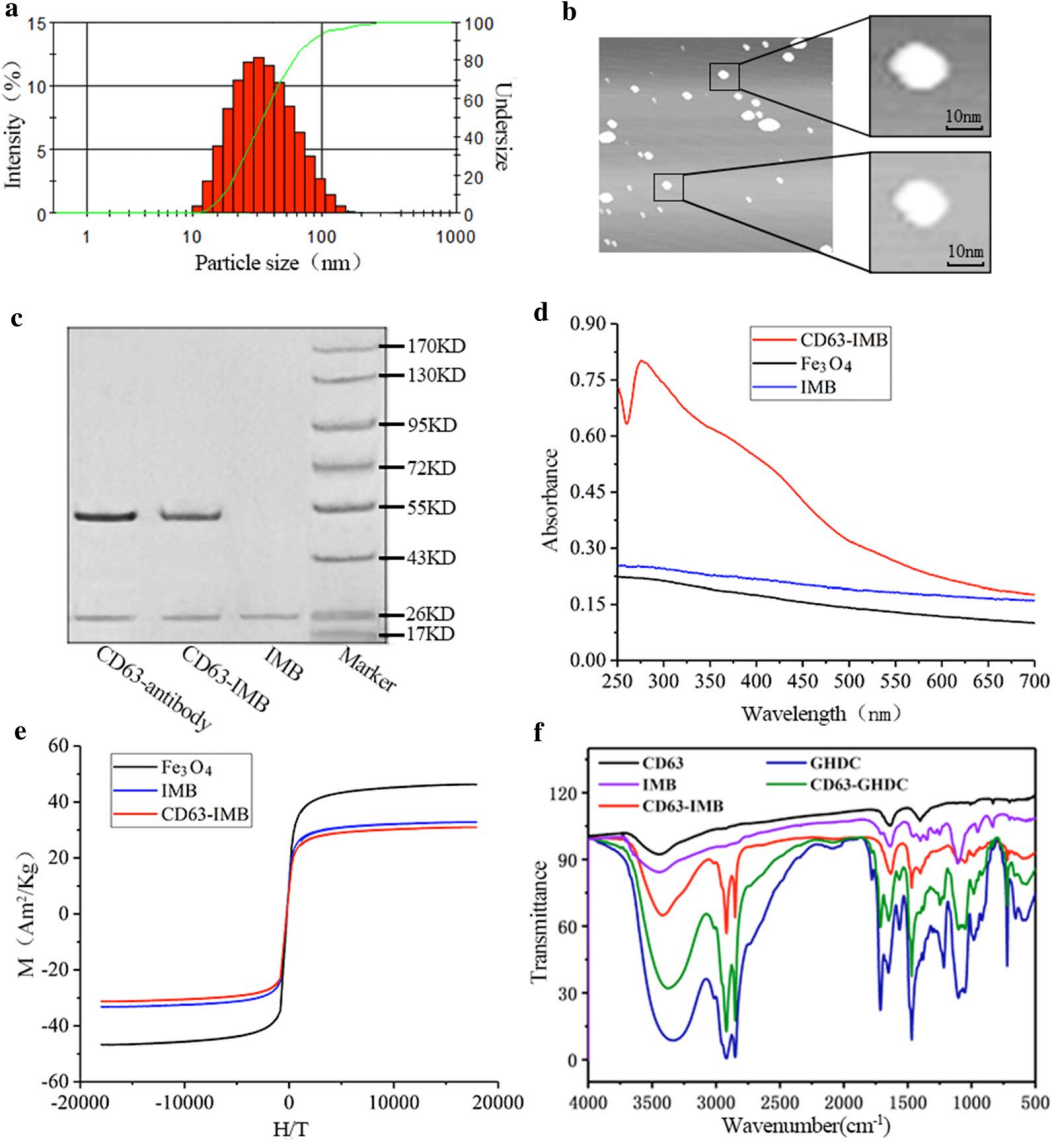
Characterization of CD63-IMB; **a** Particle size test results after 20 μL CD63-IMB diluted to 2 mL; **b** Atomic force microscope (AFM) view of 10 μL CD63-IMB; **c** Western Blot test result of CD63-IMB; **d** UV absorption after 20 μL CD63-IMBdiluted to 2 mL; **e** magnetic crystallization behavior of 2 mL CD63-IMB sample lyophilized; **f** Fourier transform infrared test result

The particle size test results of CD63 immunomagnetic beads (CD63-IMB) were shown in Fig. 1a, the average particle size was 42.53 ± 2.6 nm, the polydispersity index (PDI) was 0.105, with the particle size distribution being 10.03–118.6 nm. The test results showed that the CD63-IMB was very small in particle size and relatively narrow in particle size distribution, suggesting that the magnetic beads prepared in this study had very good stability and very good particle size distribution since the stability of solution was determined by the particle size in the solution. As shown in Fig. 1b, CD63-IMB was presented as the ball shape in different sizes under AFM, without the occurrence of agglomeration phenomenon, indicating that CD63-IMB had good stability, relatively regular shape and vesicle characteristics of liposomes. Western Blot test result displayed in Fig. 1c, CD63 and CD63-IMB have obvious protein bands around 26KD, but IMB has no bands, indicating that CD63 has been successfully coupled to CD63-IMB. The UV–Vis spectrum in Fig. 1d showed that there was a wide absorption peak at about 276 nm for CD63 antibody, while no absorption peak was observed for Fe_3_O_4_ magnetic particles or IMB, indicating that, after the CD63 antibody and the IMB were combined to form magnetic microspheres, there was also an absorption peak at 280 nm, thus the CD63 antibody was indeed bound to the surface of the magnetic spheres. The magnetization curve at the temperature of 300 K in Fig. 1e illustrated that there was no hysteresis curve at room temperature for Fe_3_O_4_ magnetic particles, and superparamagnetism was found. Figure 1e showed that the saturation magnetization of the magnetic beads was 46.27 AM^2^/kg, while there was no obvious difference in the saturation magnetization between the synthesized IMB and CD63-IMB (32.77 AM^2^/kg vs. 30.97 AM^2^/kg, *P* > 0.05), indicating that liposome materials and antibody proteins were wrapped on the surfaces of Fe_3_O_4_ magnetic particles, making the saturation magnetization of immunomagnetic microspheres/IMB decreased. The FT-IR spectra are shown in Fig. 1f, In the CD63-GHDC and CD63-IMB FT-IR spectra, new peaks appeared at approximately 2840–2930 cm^−1^, these peaks were attributed to the long carbon chain and methyl groups on the quaternary ammonium salt, representing the existence of GHDC on the CD63-GHDC and CD63-IMB.

### Isolation and identification of exosomes from clinical blood samples of GC patients by CD63-IMB

**Fig. 2:**
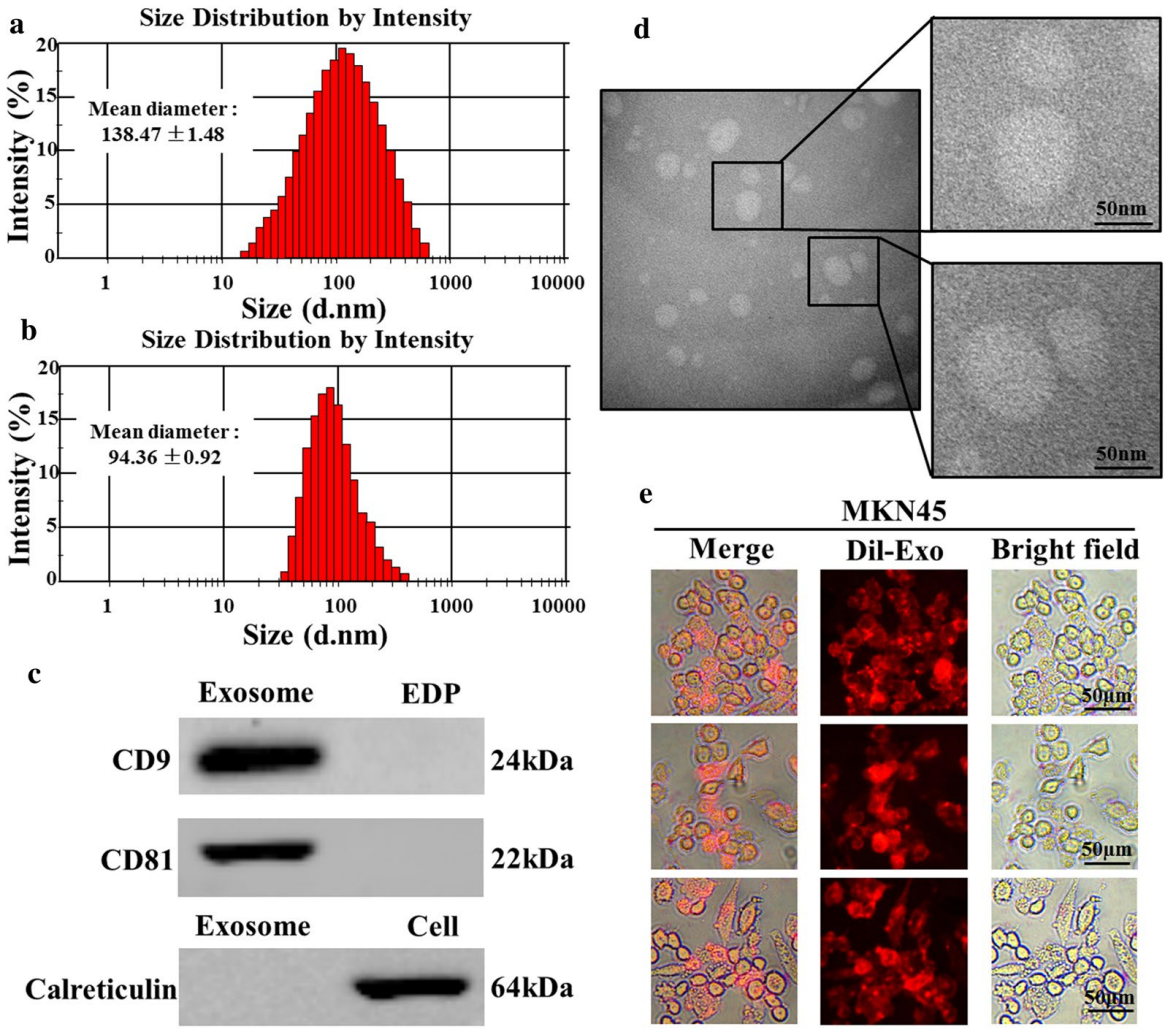
Exosome determination and cellular uptake: **a** size distribution of 2 mL exosomes enriched with CD63-IMB; **b** size distribution of 2 mL exosomes with CD63-IMB removed; **c** western blot results of exosomes, cell, No Exosome plasma(EDP); **d** Enriched 20 uL exosomes observed under TEM; **e** Take 10 μL of Dil-labeled exosomes and MKN45 cells to co-culture for 24 h, MKN-4 cellular uptake of exosomes detected by an fluorescence microscope

During nano-particle tracking analysis (NTA), Brownian motion posterior to CD63-IMB exosome is observed and the average particle size is 138.47 ± 1.48 nm (Fig. 2a). After removal of CD63-IMB, the average particle size of exosome becomes 94.36 ± 0.92 nm (Fig. 2b).In order to further verify that the vesicles are exosomes, Western Blot is performed to test CD9 and CD81, protein markers of exosomes. In the context where ER marker Calreticulin is not present in exosomes, but only in cell lysates,it is selected as a negative control (Fig. 2c). Enriched exosomes observed under TEM (Fig. 2d). It is proved by TEM based observations and NTA that the extracted exosomes are bilayer vesicles with a diameter ranging from 30 to 150 nm. Moreover, labeled exosomes are proved by fluorescence microscope to be present around cytoplasm and nucleus of the MKN-45 cell line. This indicates that MKN-45 cells are capable of effectively absorbing exosomes (Fig. 2e).

### qRT PCR results of GC cells, tissues, blood and serum exosomes

**Fig. 3:**
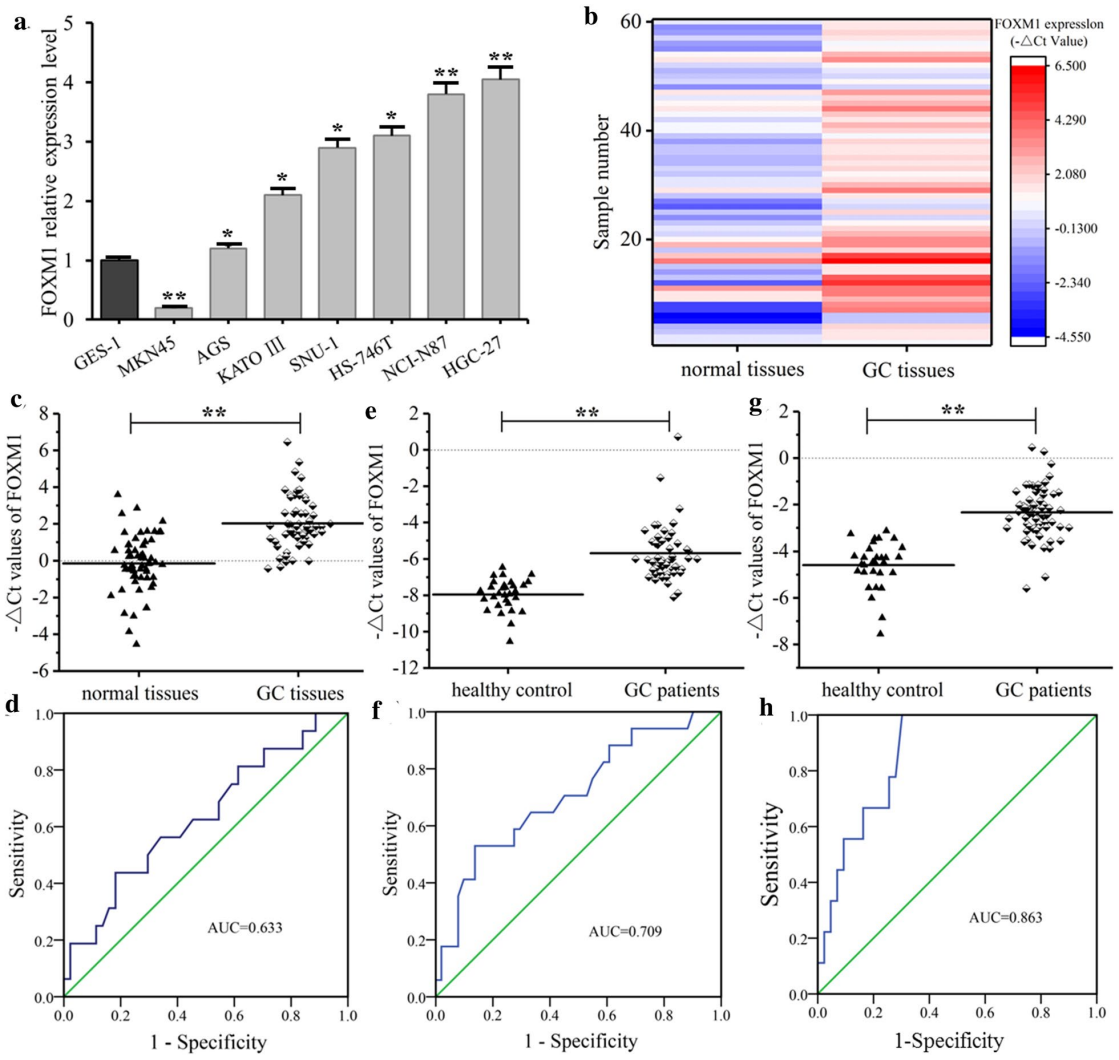
Expression levels of FRlnc1 in GC cell lines, tissues, serum and serum exosomes. **a** the expressions of FRLnc1 in GC cell lines detected by qRT-PCR (*, 0.01 < *P* < 0.05; **, *P* < 0.01); **b** scattered point distribution diagram on FRLnc1 expression levels in GC tissues (n = 60) and adjacent normal tissues (n = 60) detected by qRT-PCR; **c** hot point diagram of FRLnc1 expression levels in GC tissues (n = 60) and adjacent normal tissues (n = 60) detected by qRT-PCR: compared with adjacent normal tissues, the FRLnc1 expression level in GC tissues was significantly increased (**, *P* < 0.01); **d** ROC curve of FRLnc1 in GC patients; **e** the expression levels of FRLnc1 in 7.5 mL serum of GC patients (n = 68) and healthy controls (n = 30) detected by qRT-PCR (**, *P* < 0.01); **f** ROC curve of FRLnc1 in 7.5 mL serum of GC patients; **g** the expression levels of FRLnc1 in 7.5 mL serum exosomes of GC patients (n = 52) and healthy controls (n = 30) detected by qRT-PCR (**, *P* < 0.01); and **h** ROC curve of FRLnc1 in 7.5 mL serum exosomes of GC patients

The expressions of FRLnc1 in GC cell lines detected by qRT-PCR were shown in Fig. 3a, compared with human gastric mucosal epithelial cell line GES-1, the expressions of FRLnc1 in some GC cell lines (AGS, SNU-1, HS-746T, KATO III, NCI-N87 and HGC-27) were significantly increased, while the FRLnc1 expressions were decreased in MKN45 GC cell lines. The expressions of FRLnc1 in matched GC tissues (n = 60) and adjacent normal tissues (n = 60) were detected by qRT PCR (Fig. 3b, c), the expression of FRLnc1 in GC tissues was significantly higher than that in normal tissues. The ROC curve was drawn with the normal tissues as the control (Fig. 3d), the area under ROC curve (AUC) was 0.633 [95% confidence interval (CI) 0.471–0.795], providing a sensitivity of 52.0% and a specificity of 76.6%.

The serum FRLnc1 expressions in 68 GC patients and 30 healthy controls were detected by qRT PCR (Fig. 3e). Compared with the healthy controls, the FRLnc1 expression in serum of GC patients was significantly increased. The ROC curve was drawn, with the FRLnc1 expression in the serum of the healthy controls as the control (Fig. 3f), the AUC was 0.709 (95% CI 0.563–0.854), with a sensitivity of 74.5% and a specificity of 76.8%.

The qRT PCR was used to detect the expressions of FRLnc1 in serum exosomes of 52 GC patients and 30 healthy controls. The results showed that the serum exosomes of GC patients were rich in FRLnc1, and the expression of FRLnc1 in the serum exosomes of GC patients was significantly higher than that of the healthy controls, as shown in Fig. 3g. ROC curve drawn with the expression in the serum exosomes of the healthy controls as the control was shown in Fig. 3h, the AUC was 0.863 (95% CI 0.760–0.967), with the sensitivity of 80.6% and the specificity of 76.9%, respectively. The correlation analysis of FRlnc1 expression in serum exosomes with clinicopathological characteristics of GC patients were summarized in Table 1, the expression level of FRlnc1 in serum exosomes had no significant correlation with age, gender, tumor differentiation or other factors (all *P* > 0.05), but there were significant correlations of the expression level of FRlnc1 with LNM (*P* = 0.004) and TNM stage (*P* = 0.009).

**Table 1:** The correlation between exosomal FRlnc1 expression levels (− ΔCt) and the clinicopathological parameters of gastric cancer patients

Characteristics	Case number	Mean ± standard deviation	P	Significance
Gender				
Male	38 (73.1)	− 5.85 ± 2.55	0.285	
Female	14 (26.9)	− 6.54 ± 1.86		
Age (year)				
< 60	19 (36.5)	− 5.84 ± 1.41	0.472	
≥ 60	33 (63.5)	− 5.67 ± 2.54		
Tumor size (cm)				
< 5	31 (59.6)	− 5.14 ± 2.51	0.211	
≥ 5	21 (40.4)	− 5.98 ± 2.44		
Differentiation				
Moderate	16 (30.8)	− 5.39 ± 2.47	0.498	
Poor	36 (69.2)	− 5.85 ± 2.19		
LNM				
No	20 (38.5)	− 6.51 ± 1.85	0.004	**
N1–3	32 (61.5)	− 5.35 ± 2.77		
Invasion to veins/peripheral nerves				
No	35 (67.3)	− 5.18 ± 1.66	0.384	
Yes	17 (32.7)	− 5.49 ± 2.85		
Depth of invasion				
T1–T2	3 (5.8)	− 3.18 ± 5.24	0.651	
T3–T4	49 (94.2)	− 5.16 ± 2.44		
TNM stage				
I–II	18 (34.6)	− 5.84 ± 2.01	0.009	**
III–IV	34 (65.4)	− 5.09 ± 2.14		

*LNM* lymph node metastasis, *TNM* tumor, lymph node and metastasis; P > 0.05, the difference was not significant; 0.05 < *P* < 0.01, the difference was significant (*); *P* < 0.01, there was the difference was extremely significant (**)

### Influence of FRLnc1 knockdown on the proliferation and migration of GC cells

**Fig. 4:**
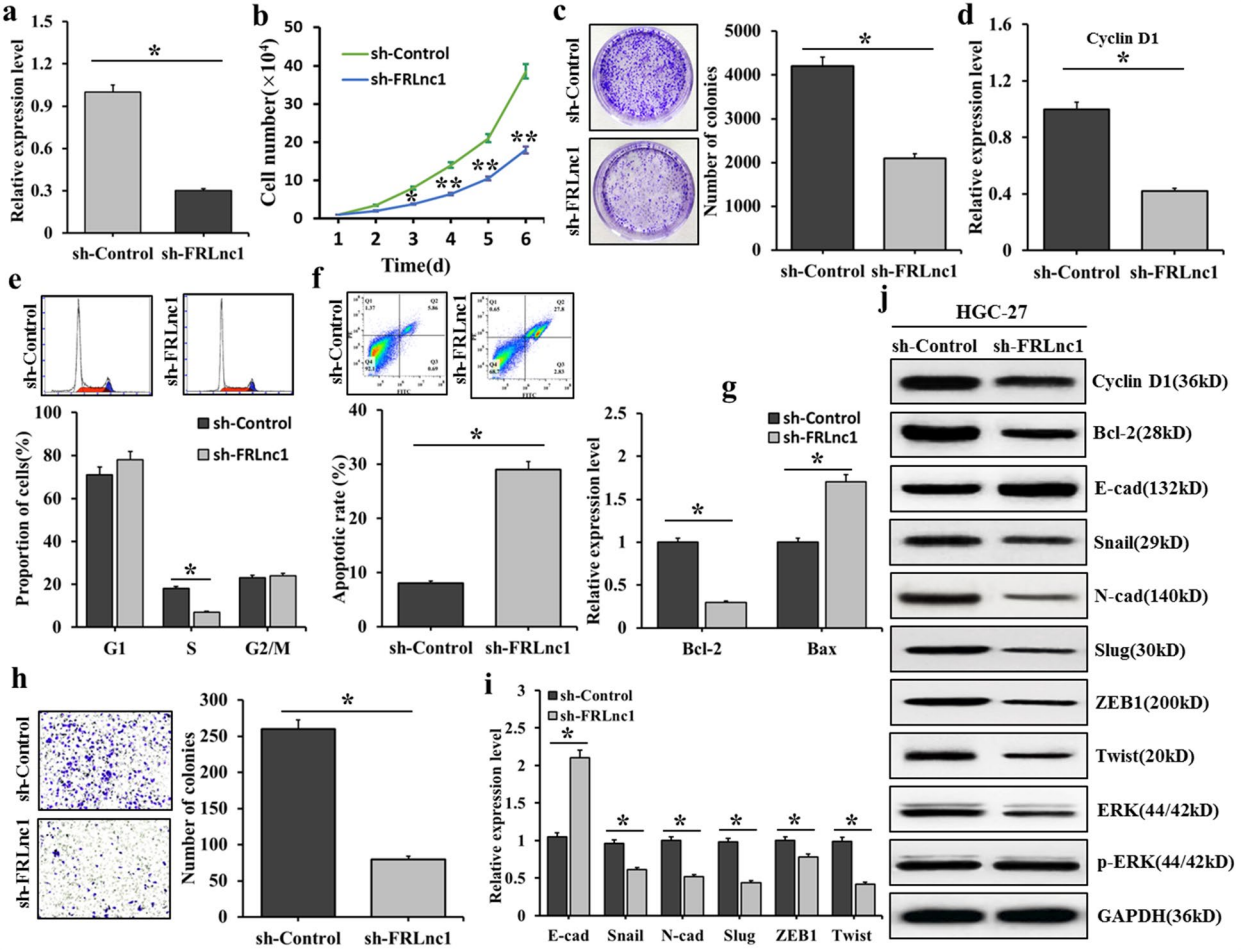
Influence of FRLnc1 knockdown on proliferation and migration of GC cells. **a** the influence of sh-FRLnc1 transfected on the expression of FRLnc1 in HGC-27 cells; **b** the growth of HGC-27 cells treated with sh-FRLnc1 transfected detected by cell growth curve; **c** the clone-forming ability of HGC-27 cells treated with sh-FRLnc1 transfected detected by cell clone-forming experiment; **d** the mRNA expression of Cyclin D1 in sh-FRLnc1 transfected HGC-27 cells detected by qRT-PCR; **e** the cell cycle of sh-FRLnc1 transfected HGC-27 cells detected by flow cytometry; **f** the cell apoptotic rates of sh-FRLnc1 transfected HGC-27 cells detected by flow cytometry; **g** the mRNA expressions of Bcl-2 and Bax in sh-FRLnc1 transfected HGC-27 cells detected by qRT-PCR; **h** the migration ability of sh-FRLnc1 transfected HGC-27 cells detected by Transwell migration test; **i** the mRNA expressions of EMT related genes in sh-FRLnc1 transfected HGC-27 cells detected by qRT-PCR; and **j** the protein expressions of cyclin D1, Bcl-2 and EMT related genes in sh-FRLnc1 transfected HGC-27 cells detected by Western blot, and the influence of exosomes containing FRLnc1 on phosphorylated ERK signaling pathways in HGC-27 cells

The results of qRT-PCR revealed that the FRLnc1 expressions were up-regulated in GC cells, tumor tissues, and serum of GC patients. An obviously up-regulated FRLnc1 expression was found in serum exosomes of GC patients, we speculated that FRLnc1 might function as an oncogene in GC. Thus, we knocked down FRLnc1 expression in GC cells. The results were shown in Fig. 4, qRT PCR results found that, compared with the control group, the expression of FRLnc1 in HGC-27 cells treated with sh-FRLnc1 transfected was markedly decreased (Fig. 4a). The growth curve of HGC-27 cells was shown in Fig. 4b, compared with the control group, the growth of HGC-27 cells was markedly decreased after sh-FRLnc1 transfected. The cell clone formation experiment found that, after sh-FRLnc1 transfected, the number of HGC-27 clone colonies markedly decreased, indicating that the FRLnc1 depletion could inhibit the proliferation of GC cells (Fig. 4c). The cell cycle experiment was shown in Fig. 4d, and the qRT-PCR results showed that the cyclin D1 mRNA level decreased in HGC-27 cells treated with sh-FRLnc1 transfected. Flow cytometry results were shown in Fig. 4e, after sh-FRLnc1 transfected, increased G1 phase cells and significantly decreased S phase cells were found. Western blot results in Fig. 4j showed that decreased cyclin D1 protein level was also observed in HGC-27 cells treated with sh-FRLnc1 transfected, indicating that FRLnc1 depletion could inhibit cycle progression of GC cells. Apoptosis results were displayed in Fig. 4f, g, compared with the control group, the cell apoptotic rates was increased significantly after sh-FRLnc1 transfected (Fig. 4f); in addition, the qRT-PCR results showed that, after exosome treatment, decreased expression of Bcl-2 and increased expression of Bax were observed(Fig. 4g); in Fig. 4j, Western blot results showed that down-regulated expression of Bcl-2 was found in HGC-27 cells treated by sh-FRLnc1 transfected, revealing that the treatment of sh-FRLnc1 transfected could promote the apoptosis of GC cells. The results of Transwell migration experiment were shown in Fig. 4h, compared with the control group, the number of migrated HGC-27 cells was markedly decreased after sh-FRLnc1 transfected treatment; in Fig. 4i, qRT-PCR was used to detect the mRNA expressions of EMT related genes, and increased expression of E-cadherin and significantly decreased expressions of N-cadherin, Slug, Snail, Twist and ZEB1 were found after sh-FRLnc1 transfected treatment; in Fig. 4j, Western blot was used to detect protein expressions of EMT related genes, and the results suggested that, up-regulated E-cadherin and down-regulated N-cadherin, Slug and Twist were found in the HGC-27 cells treated with sh-FRLnc1 transfected, indicating that the FRLnc1 depletion could inhibit the EMT of GC cells, which led to the suppression of GC cell migration. The influence of FRLnc1 depletion on ERK signaling pathways was shown in Fig. 4j, Western blot results revealed that the phosphorylated ERK (p-ERK) level was markedly decreased in HGC-27 cells treated with sh-FRLnc1 transfected, indicating that the activation of ERK signaling pathway was inhibit by the FRLnc1 depletion.

### Influence of FRLnc1 overexpression on the proliferation and migration of GC cells

**Fig. 5:**
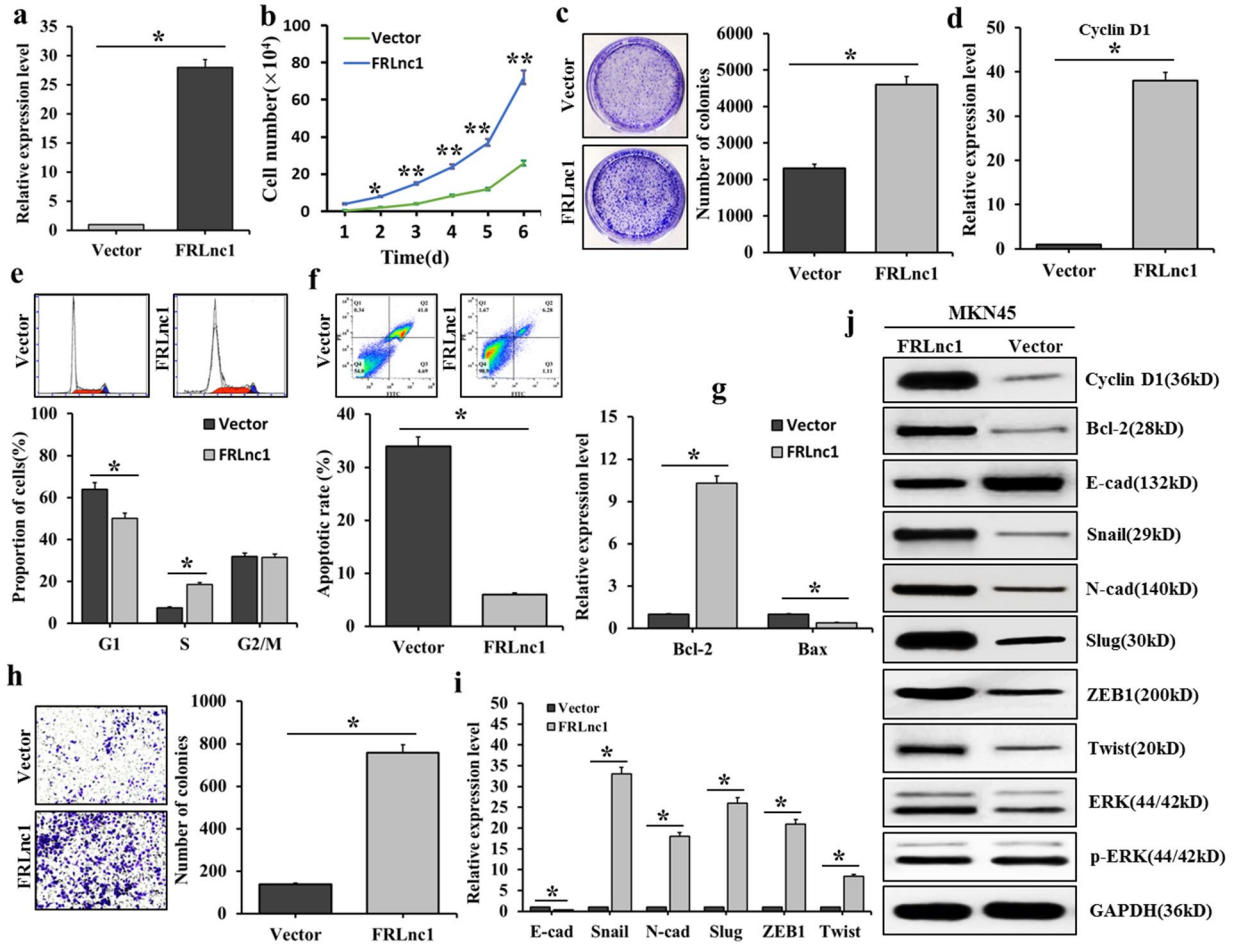
Influence of FRLnc1 overexpression on the proliferation and migration of GC cells. **a** the influence of FRLnc1 overexpression on the expression of FRLnc1 in MKN45 cells; **b** the growth of MKN-45 cells treated with FRLnc1 overexpression detected by cell growth curve; **c** the clone-forming ability of MKN45 cells treated with FRLnc1 overexpression detected by cell clone-forming experiment; **d** the mRNA expression of Cyclin D1 in FRLnc1 overexpression MKN45 cells detected by qRT-PCR; **e** the cell cycle of FRLnc1 overexpression MKN45 cells detected by flow cytometry; **f** the cell apoptotic rates of FRLnc1 overexpression MKN45 cells detected by flow cytometry; **g** the mRNA expressions of Bcl-2 and Bax in FRLnc1 overexpression MKN45 cells detected by qRT-PCR; **h** the migration ability of FRLnc1 overexpression MKN45 cells detected by Transwell migration test; **i** the mRNA expressions of EMT related genes in FRLnc1 overexpression MKN45 cells detected by qRT-PCR; and **j** the protein expressions of cyclin D1, Bcl-2 and EMT related genes in FRLnc1 overexpression MKN45 cells detected by Western blot, and the influence of FRLnc1 overexpression on ERK signaling pathways in MKN45 cells

To further demonstrate the functional roles of FRLnc1 in GC, FRLnc1 was overexpression in MKN45 cells. The results were shown in Fig. 5, qRT PCR results found that, compared with the control group, the expression of FRLnc1 in MKN45 cells treated with FRLnc1 overexpression was significantly increased (Fig. 5a). The growth curve of MKN45 cells was shown in Fig. 5b, compared with the control group, the growth of MKN45 cells was significantly enhanced after FRLnc1 overexpression treatment. The cell clone formation experiment found that, after FRLnc1 overexpression treatment, the number of MKN45 clone colonies increased significantly, indicating that the FRLnc1 overexpression could promote the proliferation of GC cells (Fig. 5c). The cell cycle experiment was shown in Fig. 5d, and the qRT-PCR results showed that the cyclin D1 mRNA level increased in MKN45 cells treated with FRLnc1 overexpression. Flow cytometry results were shown in Fig. 5e, after FRLnc1 overexpression, decreased G1 phase cells and significantly increased S phase cells were found. Western blot results in Fig. 5j showed that increased cyclin D1 protein level was also observed in MKN45 cells treated with FRLnc1 overexpression, indicating that FRLnc1 overexpression could promote cycle progression of GC cells. Apoptosis results were displayed in Fig. 5f, g, compared with the control group, the cell apoptotic rates was markedly decreased after FRLnc1 overexpression (Fig. 5f); in addition, the qRT-PCR results showed that, after FRLnc1 overexpression, increased expression of Bcl-2 and decreased expression of Bax were observed (Fig. 5g); in Fig. 5j, Western blot results showed that up-regulated expression of Bcl-2 was found in MKN45 cells treated by FRLnc1 overexpression, revealing that the treatment of FRLnc1 overexpression could inhibit the apoptosis of GC cells. The results of Transwell migration experiment were shown in Fig. 5h, compared with the control group, the number of migrated MKN45 cells was increased significantly after FRLnc1 overexpression; in Fig. 5i, qRT-PCR was used to detect the mRNA expressions of EMT related genes, and decreased expression of E-cadherin and significantly increased expressions of N-cadherin, Slug, Snail, Twist and ZEB1 were found after FRLnc1 overexpression; in Fig. 5j, Western blot was used to detect protein expressions of EMT related genes, and the results suggested that, down-regulated E-cadherin and up-regulated N-cadherin, Slug and Twist were found in the MKN45 cells treated with FRLnc1 overexpression, indicating that the FRLnc1 overexpression could promote the EMT of GC cells,, which led to the suppression of GC cell migration. The influence of FRLnc1 overexpression on ERK signaling pathways was shown in Fig. 5j, Western blot results revealed that the p-ERK level was significantly increased in MKN45 cells treated with FRLnc1 overexpression, indicating that the activation of ERK signaling pathway was enhanced by the treatment of FRLnc1 overexpression.

### Influence of FRLnc1 in GC cells-derived exosomes on the proliferation and migration of GC cells

**Fig. 6:**
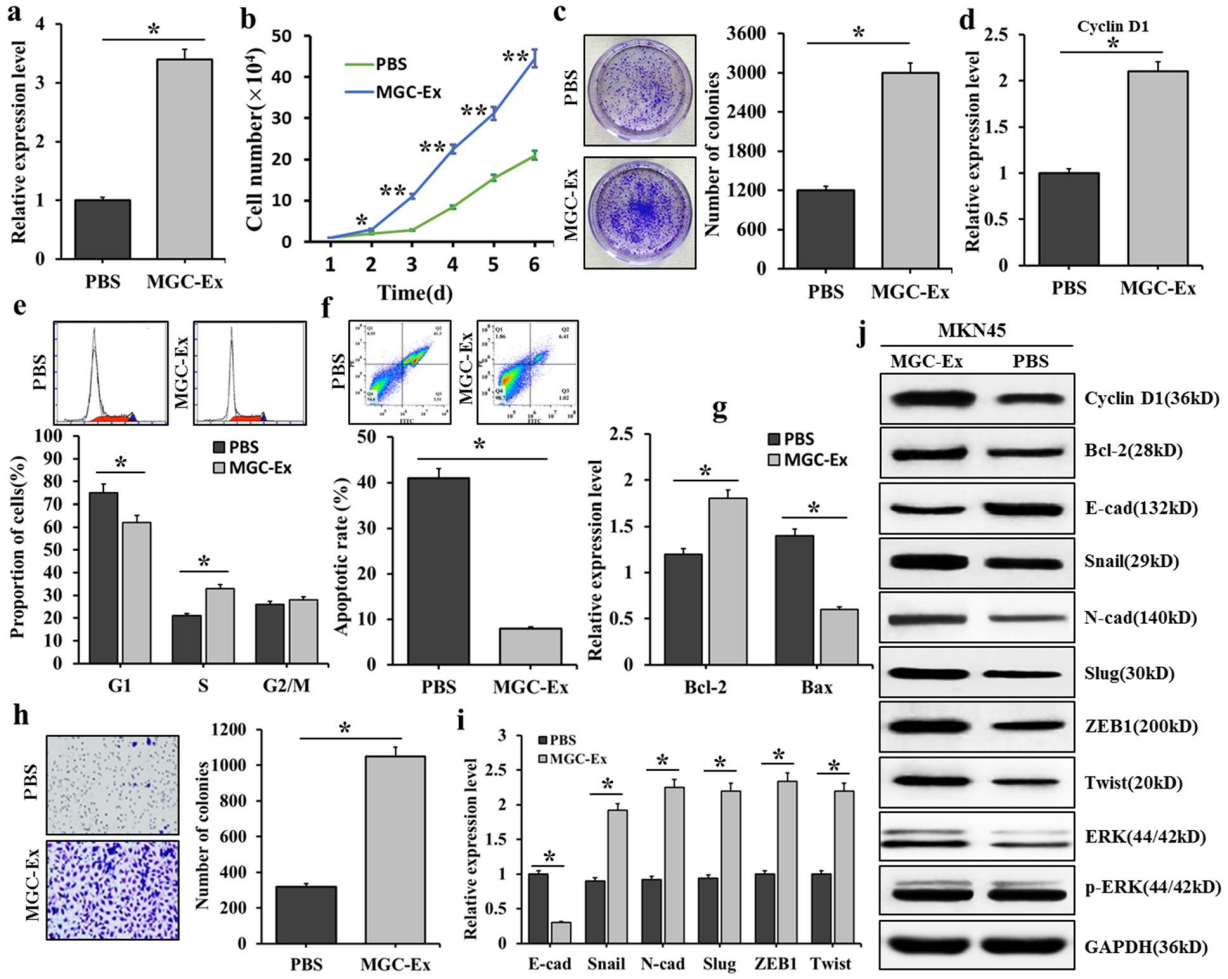
The influence of FRLnc1 in exosomes derived from HGC-27 on the proliferation and migration of GC cells. **a** the influence of exosomes containing FRLnc1 on the expression of FRLnc1 in MKN45 cells; **b** the growth of MKN-45 cells treated with exosomes detected by cell growth curve; **c** the clone-forming ability of MKN45 cells treated with exosomes detected by cell clone-forming experiment; **d** the mRNA expression of Cyclin D1 in exosome-treated MKN45 cells detected by qRT-PCR; **e** the cell cycle of exosome-treated MKN45 cells detected by flow cytometry; **f** the cell apoptotic rates of exosome-treated MKN45 cells detected by flow cytometry; **g** the mRNA expressions of Bcl-2 and Bax in exosome-treated MKN45 cells detected by qRT-PCR; **h** the migration ability of exosomes-treated MKN45 cells detected by Transwell migration test; **i** the mRNA expressions of EMT related genes in exosome-treated MKN45 cells detected by qRT-PCR; and **j** the protein expressions of cyclin D1, Bcl-2 and EMT related genes in exosome-treated MKN45 cells detected by Western blot, and the influence of exosomes containing FRLnc1 on ERK signaling pathways in MKN45 cells

The results of qRT-PCR revealed that the FRLnc1 expressions were obviously up-regulated in serum exosomes of GC patients. The exosomes were important mediators for cell signal transduction. Therefore, we determined whether exosomes-mediated transfer of FRLnc1 could affect the behaviors of the target cells. We FRLnc1 lowly-expressed MKN45 cells were treated with exosomes obtained from FRLnc1 highly-expressed HGC-27 GC cells, and then qRT-PCR, clone formation experiment, and flow cytometry and other methods were used to detect the expression levels of FRLnc1 in MKN45 cells. The results were shown in Fig. 6, qRT PCR results found that, compared with the control group, the expression of FRLnc1 in MKN45 cells treated with exosomes was significantly increased (Fig. 6a). The growth curve of MKN45 cells was shown in Fig. 6b, compared with the control group, the growth of MKN45 cells was significantly enhanced after exosome treatment. The cell clone formation experiment found that, after exosome treatment, the number of MKN45 clone colonies increased significantly, indicating that the exosomes containing FRLnc1 could promote the proliferation of GC cells (Fig. 6c). The cell cycle experiment was shown in Fig. 6d, and the qRT-PCR results showed that the cyclin D1 mRNA level increased in MKN45 cells treated with exosomes. Flow cytometry results were shown in Fig. 6e, after HGC-27 exosome treatment, decreased G1 phase cells and significantly increased S phase cells were found. Western blot results in Fig. 6j showed that increased cyclin D1 protein level was also observed in MKN45 cells treated with exosomes, indicating that exosomes containing FRLnc1 could promote cycle progression of GC cells. Apoptosis results were displayed in Fig. 6f, g, compared with the control group, the cell apoptotic rates was markedly decreased after HGC-27 exosome treatment (Fig. 6f); in addition, the qRT-PCR results showed that, after exosome treatment, increased expression of Bcl-2 and decreased expression of Bax were observed(Fig. 6g); in Fig. 6j, Western blot results showed that up-regulated expression of Bcl-2 was found in MKN45 cells treated by exosomes, revealing that the treatment of exosomes could inhibit the apoptosis of GC cells. The results of Transwell migration experiment were shown in Fig. 6h, compared with the control group, the number of migrated MKN45 cells was increased significantly after HGC-27 exosome treatment; in Fig. 6i, qRT-PCR was used to detect the mRNA expressions of EMT related genes, and decreased expression of E-cadherin and significantly increased expressions of N-cadherin, Slug, Snail, Twist and ZEB1 were found after exosome treatment; in Fig. 6j, Western blot was used to detect protein expressions of EMT related genes, and the results suggested that, down-regulated E-cadherin and up-regulated N-cadherin, Slug and Twist were found in the MKN45 cells treated with exosomes, indicating that the exosomes containing FRLnc1 could promote the EMT of GC cells, which led to the suppression of GC cell migration. The influence of exosomes containing FRLnc1 on ERK signaling pathways was shown in Fig. 6j, Western blot results revealed that the p-ERK level was significantly increased in MKN45 cells treated with exosomes, indicating that the activation of ERK signaling pathway was enhanced by the treatment of exosomes containing FRLnc1.

## Discussion

In this study, we focused on the exosome FRlnc1. Previous studies have shown that the abnormally expressed lncRNA played an important role in a variety of tumors, including GC. Specifically, Ma et al. [21] reported that the expression level of lncRNA XIST was significantly up-regulated in GC. Liu et al. [22] found that the up-regulation of lncRNA CARLo-5 was related to the poor prognosis of GC patients. Nie et al. [23] also found that lncRNA ZFAS1 was overexpressed in GC, and the increased expression of lncRNA ZFAS1 was significantly correlated to tumor size and pathological stage; and survival analysis suggested that the expression of lncRNA ZFAS1 was correlated to the prognosis of GC patients. In addition, Zhou et al. [24] demonstrated that lncRNA ZFAS1 was significantly up-regulated in 66 cases of GC tissues compared with normal tissues, and up-regulated lncRNA ZFAS1 was correlated to TNM stage, tumor invasion, LNM and tumor diameter; moreover, up-regulated lncRNA ZFAS1 was also found in plasma of GC patients, and the highly-expressed ZFAS1 in plasma was correlated to TNM Stage, LNM and distant metastasis. Our study found that the FRlnc1 expression in GC tissues was significantly higher than that in normal tissues, and the FRlnc1 expressions in serum and serum exosomes of GC patients were significantly higher than those of healthy controls. Furthermore, we found that highly-expressed FOXM1 was significantly correlated to LNM and TNM stage.

It was believed that exosome was expected to become a new molecular marker for tumors [25, 26]. The lncRNA was reported to be abundant in the exosomes obtained from different tumor cells, for example, Li found a significantly increased expression of lncRNA (LINC00152) in plasma exosomes of GC patients. The exosomes could protect the LINC00152 to enable the stable existence of LINC00152 in the blood, revealing that LINC00152 in exosomes may be a new biomarker for the diagnosis of GC [27]. Mustafa carried out detection on urine samples of 30 patients with prostate cancer and 49 patients with prostatic hyperplasia, and found that the expression of lncRNA-p21 in the urine exosomes of patients with prostate cancer was significantly increased, indicating that lncRNA-p21 in the urine exosomes could be used to distinguish prostate cancer from benign prostate diseases [28]. Exosome-mediated biomolecular transport was demonstrated to play a crucial role in regulating the biological function of receptor cells [29]. In addition, it has been shown that exosome-related lncRNA could regulate cell function, and played an important role in tumor occurrence and development [30]. Pan et al. [31] found that ZFAS1 was highly expressed in serum exosomes of GC patients, and the expression level of ZFAS1 was significantly correlated to LNM and TNM stage, and ROC curve showed that AUC was 0.837 (95% CI 0.749–0.924), with the sensitivity being 80.00% and the specificity being 75.7%, indicating that the exosomes could promote the malignant progress of GC by transporting ZFAS1. Additionally, Liu et al. [32] found that highly expressed lncRNA CRNDE-h was found in exosomes of patients with colorectal cancer, and the expression of CRNDE-h in exosomes was correlated to local LNM and distant metastasis of colorectal cancer; when the cutoff value was 0.020, the sensitivity and the specificity of CRNDE-h in serum exosomes for the diagnosis and prognosis of colorectal cancer were 70.3% and 94.4%, respectively, indicating that CRNDE-h in exosomes could be used as a noninvasive serum tumor marker for the diagnosis and prognosis of colorectal cancer. However, there were few studies on the expression and clinical value of FOXM1 in tissues or serum of tumor tissues, and the analysis of FOXM1 expression in serum exosomes of tumor patients has not been reported yet. Our study found that the expression of FRLnc1 in serum exosomes of GC patients was significantly higher than that in healthy controls, and the expression level of FRLnc1 in exosomes was significantly correlated with LNM and TNM stage; ROC curve analysis showed that the AUC of FRLnc1 in exosomes of GC patients was 0.675, with the sensitivity and the specificity being 81.6% and 75.9%, respectively, suggesting that FRLnc1 in exosomes was expected to be a potential biomarker for the diagnosis and treatment of GC, and exosomes delivery of FRLnc1 could provide a new way for the treatment of GC (Additional file 1), providing an experimental basis to further explore the biological function and mechanism of FRLnc1 in GC.

Our clinical study proved the presence of FRLnc1 in serum exosomes of GC patients, but specific biological roles of the FOXM1 in serum exosomes was not clear. Miao et al. [33] showed that FOXM1 signal pathway played an important role in the invasion of GC cells by obtaining GC EMT phenotype. In addition, Li et al. [34] found, for the first time, that TSA had the role of enhancing the sensitivity of GC cells to TRAIL through the ERK/FOXM1 pathway, revealing that FOXM1 may be used as a biomarker to predict the sensitivity of GC cells to TRAIL. Our study also found that, compared with the normal gastric mucosa cell line GES-1, FRLnc1 expressions were significantly increased in AGS, SNU-1, HS-746T, KATO III, NCI-N87 and HGC-27 GC cell lines, while decreased in MKN45 GC cell lines. Therefore, the growth and migration abilities of gastric cancer cells with FRLnc1 overexpression or knockdown in vitro were evaluated by colony formation assay and transwell migration assay. We observed that FRLnc1 knockdown could inhibit GC cell proliferation and migration while FRLnc1 overexpression had the opposite effects. Mechanistically, FRLnc1 knockdown induced cell cycle arrest and cell apoptosis and suppressed EMT in GC cells. In addition, the exosomes containing FRLnc1 were further isolated from GC HGC-27 cells, and MKN45 GC cells with low expression of FRLnc1 were treated with exosomes, in order to further explore the role of FRLnc1 in the occurrence and development of GC. We found significantly increased expression of FRLnc1 in MKN45 cells after exosomes treatment, and FRLnc1 in exosomes derived from GC cells could enhance the proliferation and migration of GC cells. The main conclusions of this study were from in vitro experiments, thus in vivo experiments were needed to further confirm our findings. The specific molecular mechanisms of the role of FRLnc1 in GC were not fully understood, which needed to be further explored by in-depth study. In addition, the clinical value of serum exosome FRLnc1 as a molecular marker of GC needed to be further verified by expanding the sample size. Moreover, the relationship between serum exosome FRLnc1 and the prognosis of GC needed to be further investigated by in-depth study. Solving the above-mentioned limitations could help us fully understand the role of FRLnc1 in GC, which was of great significance to use lncRNA as a new target for the diagnosis and treatment of GC.

## Conclusion

In conclusion, the prepared CD63-IMB could effectively isolate serum exosomes, significantly up-regulated expressions of FRlnc1 were found in cells, tumor tissues, serum and serum exosomes of GC patients, and the high expression of FRlnc1 in exosomes was suggested to be significantly correlated to LNM and TNM stage. The GC cell-derived exosomes could promote the growth and metastasis of GC by transporting FRlnc1, indicating that exosome FRlnc1 may be a potential biomarker for the diagnosis and treatment of GC, and the delivery of FRlnc1 by exosomes may provide a new way for the treatment of GC.

## Methods

### Sample collection and ethical statement

In the study, samples were collected from GC patients receiving surgical treatment from January 2017 to November 2020 in our hospital, including tumor tissue samples (n = 60) and adjacent normal tissue samples (adjacent normal tissues were collected 5 cm away from the tumor tissue edges) (n = 60), and all the collected tissue samples were immediately put into liquid nitrogen for rapid freezing and stored at − 80 °C for further RNA extraction. Peripheral venous blood samples (7.5 mL) were separately collected from 68 GC patients and 30 healthy volunteers, and all the blood samples were stored at 4 °C for transportation, followed by processing within 72 h. Clinical information was collected from all patients enrolled in the cohort. The study was approved by the Ethics Committee of Tongji Hospital of Tongji University (2020-KYSB-094), and informed written consent was obtained from all patients.

### Materials and instruments

Human GC cell strains, including AGS, SNU-1, HS-746T, KATO III, NCI-N87, HGC-27 and MKN45, were obtained from Shanghai Cell Bank of Chinese Academy of Sciences, and a normal human gastric epithelial cell strain (GES-1) was obtained from Shanghai Gefan Biotechnology Co., Ltd.. RPMI 1640 medium was purchased from Invitrogen; DMEM/F-12 medium was from Thermo; DMEM medium, fetal bovine serum (FBS) and trypsin were from Gibco; exosome extraction kit was from SBI, USA; Trizol kit, ultrapure RNA extraction kit, RNA reverse transcription kit and the Real-time PCR detection kit were from Beyotime Biotechnology Co., Ltd.; cell cycle detection kit and cell apoptosis detection kit were from Fcmacs Biotech Co., Ltd. (Nanjing); ZFES 1 shRNA, ZFES 1 overexpression plasmid and HB-TRLF-1000 LipoFilter transfection kit were from Hanbio Biotechnology Co., Ltd. (Shanghai); CD63 antibody was from eBioscience (USA); extracellular signal-regulated kinases (ERK), phosphorylated ERK (p-ERK), Cyclin D1, Bcl-2, E-cadherin, N-cadherin, Slug, Twist and GAPDH antibodies were from Cell Signaling Technology, Inc (CST) (USA); hexadecyl-quaternized carboxymethyl chitosan (HQCMC), ferroferric oxide (Fe_3_O_4_) nanoparticles and magnetic separation rack were from Huzhou Lieyuan Medical Laboratory; 1,2-dioleoyl-sn-glycero-3-phosphocholine (DOPC) and dimethyloctadecyl-epoxypropane-ammonium chloride (GHDC) were from Jukang (Shanghai) Biotechnology Co., Ltd.; cholesterol (Chol), methylene chloride and other common reagents were purchased from China National Pharmaceutical Group Corporation; TALOS F200X electron microscope was from Thermo Fisher Scientific; Zetasizer Nano ZS was from Malvern Instruments Inc, Malvern, UK; 7407 Vibrating Sample Magnetometer (VSM) was from Lake Shore; ND-1000 NanoDrop was from Calibre (Beijing) Technology Development Co., Ltd.; and quantitative PCR instrument was from Bio-Rad, USA.

### Material preparation

**Fig. 7:**
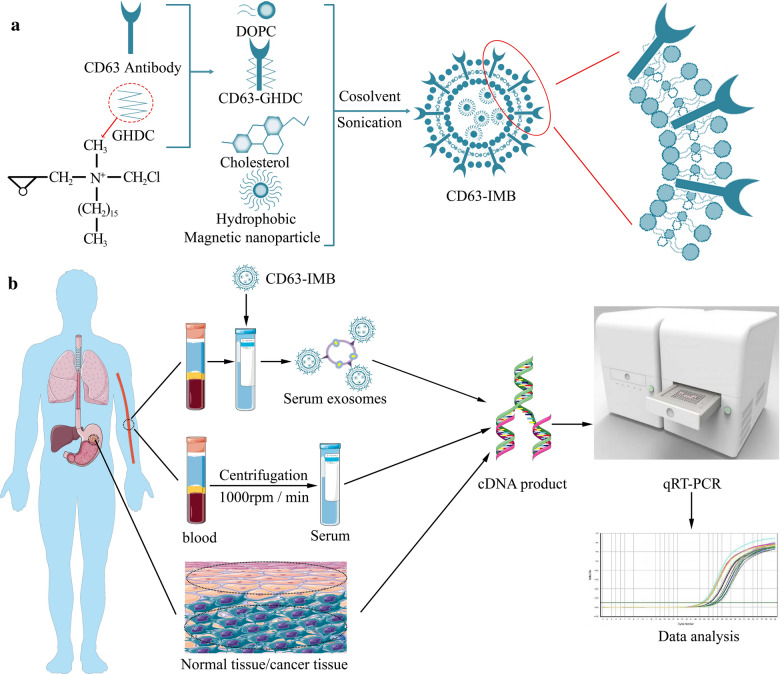
Preparation flow chart and Flow chart of clinical sample detection. **a** The preparation process of CD63-IMB. Fe3O4 nanoparticles are encapsulated in liposomes under ultrasonic conditions through various lipid materials, and CD63 antibodies are conjugated on the surface to prepare CD63-IMB; **b** Gc clinical sample detection procedure: tissues and blood of GC patients were collected, and the expression of FRLNC1 in tissues, blood and serum exosomes was detected by qRT-PCR

CD63 is called lysosomal-associated membrane protein 3, which is a specific marker protein on the surface of exosomes. After incubating with the magnetic beads coated with anti-label antibody (CD63 antibody) and the exosomal vesicles, the exosomes can be adsorbed and separated [35]. The preparation of CD63-IMB included the following steps: co-dissolving DOPC (10 mg), Chol (10 mg), HQCMC (5 mg) and Fe_3_O_4_ (10 mg) nanoparticles in chloroform, add 6 mL of distilled water, and use a probe-type Ultrasonic instrument to ultrasonically oscillates the mixed solution with a power of 27% and a total time of 6 min (each ultrasonic oscillation interval is 1 s, and the interval is 2 s), and the mixed solution is completely emulsified at a temperature of 25 ℃, and rotary evaporation for 30 min to obtain immunomagnetic beads (IMB); and dissolving the GHDC (10 mg) in absolute ethanol, simultaneously adding a CD63 antibody (60 µg), and carrying out co-incubation to obtain CD63-GHDC, adding the CD63-GHDC into the IMB, followed by reacting for 24 h, thereby obtaining CD63-modified IMB, named as the CD63-IMB (Fig. 7a). Separate the CD63-IMB with a magnet and wash it to remove excess free antibody. Resuspend the CD63-IMB in 2 mL PBS and store at 4 °C [36, 37].

### CD63-IMB characterization test

The CD63-IMB characterization test comprised the following steps [38, 39]: taking 20 μL of CD63-IMB solution, diluting the solution to 2 mL by pure water, and detecting the particle size by the Zetasizer Nano ZS. Sample was carried out preparation on a mica sheet by using 10 μL of CD63-IMB, and observing the shape by atomic force microscope (AFM) after drying. Weighing 20 μL of CD63-IMB solution, and carrying out CD63 protein detection by Western blot (WB). 2 mL of CD63-IMB sample go through freeze-drying, and detecting the magnetic property through VSM. Weighing 20 μL of CD63-IMB solution to be diluted to 2 mL with purified water, and carrying out UV detection by UV–Vis spectroscopy. Fourier transform infrared(FT-IR) spectroscopy were recorded on a Bio-Rad FTS 6000 spectrometer with KBr pellets at room temperature.

### Isolation and identification of exosomes in clinical blood samples of GC patients by CD63-IMB

Anticoagulated blood samples (7.5 mL) were collected from the GC patients, and then centrifuged at 1000 rpm/min for 10 min. The supernatant was carefully placed in an EP tube. CD63-IMB (30 μL) was added into the tube, followed by incubation at room temperature for 30 min (mixing every 10 min). The EP tube was placed in a magnetic separation rack for separation for 5 min, the supernatant was removed, obtain the exosomal solution captured by magnetic beads. Trypsin (2 mL) was added into the EP tube, add trypsin inhibitor (2 mL) after digestion at 37 °C for 10 min, the tube was vortexed for 5 min and immediately placed in the magnetic separation rack for 10 min, and collect the supernatant, thereby obtaining exosome suspension.

The collected exosome suspension (20 μL) was put onto a grid coated with polyvinyl acetate/carbon in a dropwise manner for filtering, followed by standing for 5 min, residual liquid at the edge of the grid was slightly sucked by using filter paper, 3% (W/V) phosphotungstic acid was dropped onto a film, the grid was inverted on the film for dyeing for 1 min, and the film was placed under the TEM for observation after the film was dried.

Cell lysate containing protease inhibitor PMSF was added into the captured exosome solution according to the ratio of 1: 1, the mixture was subjected to shaking and uniform mixing, followed by standing on ice for 30 min, and protein loading buffer was added (1: 4), followed by uniform mixing and boiling water bath for 15 min; 10% SDS-PAGE electrophoresis gel was prepared, and protein samples and a protein Marker were added into tooth comb-formed gel holes for gel electrophoresis; after gel electrophoresis, proteins were transferred to a PVDF membrane at 350 mA for 2 h, so as to transfer the target band to the PVDF membrane; the PVDF membrane was placed in 5% skim milk for blocking the bands at room temperature for 2 h, followed by incubating with the rabbit anti-human CD9 antibody, the rabbit anti-human CD81 antibody and the GAPDH internal reference antibody, respectively, and then placed in a refrigerator at 4 °C overnight; the membrane was rinsed with TBS/T for three times, incubated with the secondary antibody at 37 °C for 2 h, rinsed with TBS/T for three times, and the electrochemiluminescence (ECL) system was used for detection.

The exosome sample solution (10 μL) was diluted 10 × with PBS for NanoSight analysis.

Dil dye was added into the exosome solution (10 μL), followed by incubating at 37 °C for 30 min, PBS was added to dilute the Dil-labeled exosome solution to 10 mL, followed by centrifuging at 1000*g* for 30 min, the Dil-labeled exosome solution was added into MKN45 cells, followed by incubating at 37 °C for 24 h, the culture medium was discarded, the MKN45 cells were washed with PBS for 2 times, and imaging analysis was performed with a fluorescence microscope.

### RNA extraction and qRT-PCR on GC cells, tissues, blood and serum exosomes

SNU-1, NCI-N87, HGC27 and MKN45 cells were cultured in RPMI 1640 medium containing 10% FBS, HS-746T cells were cultured in DMEM medium containing 10% FBS, KATO III cells were cultured in IMDM-H medium containing 10% FBS,AGS cells were cultured in DMEM/F-12 medium containing 10% FBS, and the cells were cultured in a 5% CO_2_ incubator at 37 °C. The culture medium was changed every 3 days, the cell growth state was closely observed, and cell passage could be performed when the cell density reached 80%.

In order to study the FRlnc1 expressions in GC cells, tissues, blood and serum exosomes, qRT-PCR was separately carried out on GC cell lines, primary GC tissues and adjacent normal tissues, blood samples of healthy controls and GC patients and serum exosomes of healthy controls and GC patients. The detection flow chart was shown in Fig. 7b.

The cDNA products in cells, tissues, blood and serum exosomes were obtained by Trizol reagent, RNA extraction kit and RNA reverse transcription kit according to the manufacturer’s instructions, and stored at − 80 °C for future use. System preparations were performed according to the qRT-PCR Mixture reagent specifications. With GAPDH as the internal reference gene, primer sequences were as follows: FRLnc1: forward primer, 5′-ATGCGTGATTGCAGTCTCTG-3′; reverse primer, 5′-TCTTGCAATATTTCCTGTGA-3′; GAPDH: forward primer, 5′-GTCAACGGATTTGGTCTGTATT-3′; reverse primer, 5′-AGTCTTCTGGGTGGCAGTGAT-3′. Cell and tissue qRT-PCR system: 10 μL 2 × Mixture, 0.3 μL upstream and downstream primers (10 μmol/L), and 1.5 μL template cDNA, with a total volume of 20 μL by adding 7.9 μl RNase-free water; serum qRT-PCR system: 10 μL 2 × Mixture, 0.3 μL upstream and downstream primers (10 μmol/L), and 2.0 μL template cDNA, with a total volume of 20 μL by adding 7.4 μL RNase-free water; and exosome qRT-PCR system: 10 μL 2 × Mixture, 0.3 μL upstream and downstream primers (10 μmol/L), and 2.5 μL template cDNA, with a total volume of 20 μL by adding 6.9 μL RNase-free water. After evenly mixing and instantaneously centrifuging, qRT-PCR was carried out with the use of Bio-Rad qRT-PCR instrument, with the pre denaturation procedure being 94 °C for 10 min and the amplification procedure being 94 °C for 30 s, 55 °C for 30 s and 72 °C for 30 s, with a total of 40 cycles. GAPDH was used as the internal reference gene, and the results were analyzed by relative quantitative 2^−ΔΔCt^ method.

### Influence of FRLnc1 on proliferation and migration of GC cells

#### Gene silencing and overexpression

The sh-FRLnc1 directed against FRLnc1 and FRLnc1-over-expressing plasmid were purchased from General biosystems (Anhui) Co., Ltd. The sequences of sh-FRLnc1 and the shRNA control (sh-Ctrl) were shown (sh-FRLnc1: sense 5-CAAGAUUAAAUGCCUAAGAdTdT-3); sh-control: sense 5-UUCUCCGAACGUGUCACGUdTdT-3). HGC-27 and MKN-45 cells were grown in 6-well plates (2 × 10^6^/well) and transfected for 36 h using HB-TRLF-1000 LipoFiter transfection reagent.

#### Cell growth curve measurement

Cell transfection was carried out with the use of Lipofiter liposome transfection reagent. After 36 h of transfection, cells were digested into single cell suspension by trypsin, inoculated into 24-well plates (10,000 cells per well), and counted every 24 h after digestion for 6 consecutive days. Operations were repeated 3 times per well. The cell growth curve was drawn with the time as the X-axis and cell number as the Y-axis.

#### Cell clone-formation experiment

The transfected cells were digested by trypsin, inoculated into a 6-well plate (1000 cells per well) and cultured in a 37 °C constant temperature incubator, and the culture medium was changed every two days. After culture for about 10 days, the 6-well plate was taken out when the cell clone could be observed by naked eyes; and the culture supernatant was discarded; the cells were washed twice with PBS, fixed with 4% polyformaldehyde for 30 min, subjected to crystal violet staining for 15 min, and washed twice with PBS; each experiment was repeated three times per well. The cell clones were recorded by camera and counted under microscope.

#### Transwell migration experiment

The transfected cells were digested into single cell suspension by trypsin, inoculated in serum-free medium, and placed in the upper chamber of Transwell plate, the lower chamber was medium containing 10% FBS, cells were cultured in a constant-temperature cell incubator at 37 °C for 36 h, the Transwell chamber was removed, the upper chamber solution was completely sucked, non-migrated cells on the inner surface of the chamber were carefully and gently wiped off with a cotton swab, and the chamber was placed in 4% paraformaldehyde fixation fluid for cell fixation for 30 min; cells were stained with crystal violet for 15 min., and washed with PBS for 3 times; each experiment was repeated for 3 times per well. After drying, the cells were photographed and recorded under the microscope, and the number of transferred cells was counted.

#### Cell cycle determination

The transfected cells in the logarithmic growth stage were collected, and digested into single cell suspension with trypsin; the cell suspension was transferred into a new centrifuge tube, and centrifuged (300*g*, 5 min); cell precipitations were collected, washed twice with pre-cooled PBS, and resuspended with pre-cooled PBS twice to make the cells fully suspended into single cells, pre-cooled anhydrous ethanol was added, the resuspended cells were pipetted and evenly mixed, fixed overnight at 4 °C, centrifuged at 800 rpm for 10 min, rinsed with PBS A once, and additionally centrifuged to obtain the cell precipitates; RNase A was added into the cell precipitates, followed by water bath at 37 °C for 30 min; the cell precipitates were subjected to staining at room temperature away from light with 100 μg/mL propidium iodide (PI) for 30 min. The cells in different cell cycle phases were counted by flow cytometry with standard procedures.

#### Cell apoptosis detection

The transfected cells were digested into single cell suspension by trypsin. The cell suspension was pipetted into a new centrifuge tube, and centrifuged at 4 °C, 300*g* for 5 min; the cell precipitates were collected, washed with pre-cooled PBS twice and centrifuged at 4 ℃, 800 rpm for 10 min; 1 × Binding buffer (4 mL binding buffer + 12 ml deionized water) was prepared, an appropriate amount of 1 × Binding buffer was added to resuspend the cells, with the concentration adjusted to 1 × 10^6^/mL; 100 μL cells were suspended in 5 mL flow tube, 5 μL Annexin Alexa Fluor 647 and 10 μL PI were added successively, the materials were gently and evenly mixed, followed by standing at room temperature for 15 min, with the need of keeping the mixture away from light, 400 μL PBS were added, followed by uniform mixing; and cell apoptosis detection was carried out within 1 h with the use flow cytometer, and apoptotic cells were counted.

#### Extraction of total protein from cells

Cell culture supernatant was discarded, cells were washed with PBS twice, an appropriate amount of cell lysate (including protease inhibitor) was added into the culture plate, the materials were evenly mixed, and the mixture was shaken for 5 min by a shaker. Cells were scraped off with a cell scraper, and transferred to the Eppendorf tube with a pipette, followed by even blending, the Eppendorf tube was placed on ice, violently shaken for 1 min every 10 min and centrifuged (4 °C, 12,000*g*, 15 min) after four times of shaking, after centrifugation, the supernatant was pipetted into a new Eppendorf, the extracted total protein concentration was measured with the use of the NanoDrop 1000 spectrophotometer, and the data was recorded. The sample loading buffer was added into to the proteins to be evenly mixed, boiled in boiling water for 10 min and stored in a − 20 °C refrigerator.

#### Western blot

The 12% sodium dodecyl sulfate-polyacrylamide gel electrophoresis (SDS-PAGE) running gel was prepared, the protein samples and the protein Marker were added into the tooth comb-formed gel holes, and the electrophoresis was ended when the indicator bands were fully separated and the target bands could be clearly obtained (80 V constant voltage, and the voltage was changed to 100 V when the sample entered the separation gel); after electrophoresis, the gel was removed, the PVDF membrane was covered on the gel, membrane transferring was carried out at 350 mA constant voltage for 2 h to transfer the target bands on the gel to the PVDF membrane, with the need of carrying out membrane transferring process on ice. At the end of membrane transferring, 5% skim milk was used to block the membrane at room temperature for 2 h, followed by incubation with the corresponding primary antibody in the − 4 °C refrigerator overnight, and the membrane was washed with TBS/T for 3 times, incubated with the secondary antibody at 37 °C for 2 h, washed with TBS/T for 3 times; and ECL chemiluminescence system was applied for detection.

### Influence of GC cell-derived exosome FRLnc1 on proliferation and migration of GC cells

Cells were centrifuged for 16 h to remove the exosomes, HGC-27 cells were cultured in the exosome-removed culture medium, HGC-27-derived exosomes (100 μg/mL) and PBS were added to MKN-45 cells, respectively, and then the mixture was cultured in RPMI 1640 medium. The cell culture and detection analysis method was the same as above.

### Statistical analysis

SPSS 22.0 was used for statistical analysis, t-test or one-way ANOVA was used to compare the differences between the experimental groups, Pearson χ^2^ test was used to analyze the correlations between the FRLnc1 expression and clinicopathological data, and ROC curve was established to analyze the clinical diagnosis value, with *P* < 0.05 being statistically different (*, *P* < 0.05 and **, *P* < 0.01).

## Supplementary Information

**Additional file 1. **Compare the results of this research with related research.

## Data Availability

All data generated and analyzed during this study are included in this published article.
