# Supplementary material for: Expression and mechanism of exosome-mediated A FOXM1 related long noncoding RNA in gastric cancer
Source: J Nanobiotechnology. 2021 May 10;19:133. doi: 10.1186/s12951-021-00873-w (PMC8111998; doi:10.1186/s12951-021-00873-w)
Supplement: Supplementary file 1 — Additional file 1. Compare the results of this research with related research. [file 12951_2021_873_MOESM1_ESM.docx]

Additional file

Additional file 1. Compare the results of this research with related research

| Tumor type | exosomes lncRNA | Sensitivity | Specificity | Conclusion |
| --- | --- | --- | --- | --- |
| Stomach cancer | ZFAS1 | 80.00% | 75.7% | Indicating that the exosomes could promote the malignant progress of GC by transporting ZFAS1 [32]. |
| Colon cancer | CRNDE-h | 70.3% | 94.4% | Indicating that CRNDE-h in exosomes could be used as a noninvasive serum tumor marker for the diagnosis and prognosis of colorectal cancer [33]. |
| Stomach cancer | FOXM1 | 80.6% | 76.9% | Suggesting that FRLnc1 in exosomes was expected to be a potential biomarker for the diagnosis and treatment of GC, and exosomes delivery of FRLnc1 could provide a new way for the treatment of GC. |
